# Orthotic Bracing or Minimally Invasive Surgery? A Summary of 767 Pectus Carinatum Cases for 9 Years

**DOI:** 10.1155/2021/6942329

**Published:** 2021-02-19

**Authors:** Ziyin Shang, Chun Hong, Xianlun Duan, Xiangyong Li, Yuan Si

**Affiliations:** ^1^Department of Pediatric Thoracic Surgery, Guangdong Women and Children Hospital, No. 521, Xingnan Road, Panyu District, Guangzhou 511400, China; ^2^Department of Thoracic Surgery, Anhui Province Children's Hospital, No. 39, Wangjiang East Road, Baohe District, Hefei 230051, China; ^3^Department of Infectious Diseases, The Third Affiliated Hospital of Sun Yat-sen University, No. 600 Tianhe Road, Tianhe District, Guangzhou 513610, China; ^4^Center for Brain Diseases, The Third Affiliated Hospital of Sun Yat-sen University, No. 600 Tianhe Road, Tianhe District, Guangzhou 513610, China

## Abstract

Orthotic bracing and minimally invasive surgery are currently the treatment methods for pectus carinatum. We present our experience with the advantages, method selection criteria, and precautions for both treatment methods. A total of 767 pediatric patients (596 boys and 171 girls) with pectus carinatum were retrospectively analyzed. All of them received orthotic bracing, and 108 pediatric patients received minimally invasive surgery, achieving good outcomes. Among the 767 pediatric patients, 644 obtained satisfactory chest appearance through orthotic bracing, with a success rate of 84.0%. Younger pediatric patients had better orthotic outcomes. Among the 123 failure cases, 108 pediatric patients underwent minimally invasive surgery as the treatment. Seventy-six pediatric patients with simple chondrogladiolar prominence underwent a minimally invasive sternal depression procedure, whereas 32 pediatric patients with complex chondromanubrial prominence underwent surgery. All 108 pediatric patients successfully completed the surgery. The operation time was 112.8 ± 23.5 min, and the average length of hospital stay after the surgery was 7 days. A follow-up was conducted for at least 3 months and up to 3 years. The orthotic effect was satisfactory. For younger pediatric patients with pectus carinatum, noninvasive orthotic bracing treatment should be considered first. For older pediatric patients, the failure rate of the bracing was higher, and the outcomes were often unsatisfactory. Especially for patients over 15 years old, minimally invasive sternal depression may be the preferred treatment for pectus carinatum.

## 1. Introduction

Pectus carinatum is a common chest wall deformity seen in children. It influences the chest appearance of pediatric patients and often leads to adverse effects on their growth, development, and mental health. Most of the affected children lack confidence and are reluctant to bathe in public bathrooms, swim, or take part in outdoor activities. To cover their protruding chest, they often bend while walking and sitting. Abnormal posture and lack of training also aggravate the deformity. Thoracic deformity of pectus carinatum is likely to present with the trend of progressive aggravation with age. Thus, after the diagnosis of pectus carinatum is confirmed, patients should be actively treated. The Ravitch sternal depression procedure and its modifications are traditional surgical techniques for the treatment of pectus carinatum [1]; however, since the procedure is highly invasive, it is often not accepted by parents and pediatric patients. Currently, the main treatment methods for pectus carinatum are noninvasive orthotic bracing and minimally invasive sternal depression surgery [2–4], and their orthotic effects are significant. These noninvasive and minimally invasive treatment methods are also deeply welcomed by parents and pediatric patients.

Through a retrospective analysis of 767 pediatric patients with pectus carinatum treated by orthotic bracing and a minimally invasive sternal depression procedure from August 2010 to August 2019, we concluded the advantages and selection of treatment methods between these two treatments and investigated the treatment experience and precautions.

## 2. Materials and Methods

**Figure 1:**
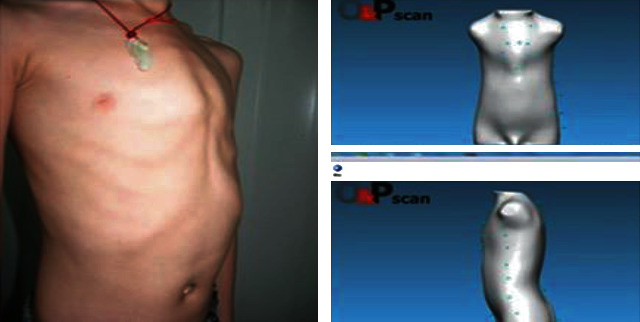
Three-dimensional chest wall scanning and reconstruction for pectus carinatum.

**Figure 2:**
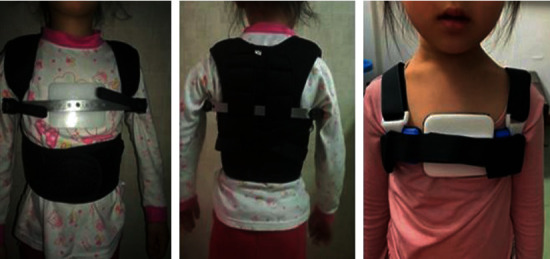
Wearing of orthotic bracing for pectus carinatum.

From August 2010 to August 2019, a total of 767 pediatric patients with pectus carinatum were treated in the Department of Thoracic Surgery at Anhui Provincial Children′s Hospital. The patients and their parents agreed to participate in the study. There were 596 boys and 171 girls. The median age was 8 years and 9 months (age range, 26 months to 17 years). Before the treatment, all pediatric patients underwent frontal and lateral chest X-ray examination to exclude chest wall deformities caused by other cardiopulmonary diseases or anterior mediastinal tumors. Since there were differences in the thorax size among the pediatric patients, the transverse and anteroposterior diameters of the chest at the highest point of the sternum bulge were measured in each patient. Each patient underwent three-dimensional chest wall scanning and reconstruction (Figure 1), as it can accurately record the pattern of chest wall deformity without radiation [5, 6]. According to the scanning metrics, a suitable orthotic brace was fabricated for each pediatric patient. The pediatric patients were requested to wear the orthotic braces every day for more than 12 h, and reexaminations were carried out every 2 months. The orthotic pressure was adjusted based on the treatment effect. Usually, the older children needed longer treatment time. The average brace treatment time was 0.5–1 year. The chest of younger pediatric patients was flat after half a year. After treatment, the treatment time was half a year. For older pediatric patients, half a year is often not enough, and they need a year or longer. If the sternum protrusion did not improve after 1 year of treatment or longer, it was judged as a failed orthopedic treatment (Figure 2).

Among the pediatric patients in whom orthotic bracing failed, 108 underwent minimally invasive surgery. There were 85 boys and 23 girls. The median age was 13.5 years (age range, 9 years and 11 months to 17 years). There were 76 cases of simple chondrogladiolar prominence type of pectus carinatum and 32 cases of complex chondromanubrial prominence type of pectus carinatum. Among them, there were 29 cases of obvious Harrison groove depression, two cases of pouter pigeon breast with pectus excavatum, and one case of neurofibromatosis. Before the surgery, all pediatric patients underwent a routine blood test, frontal and lateral chest X-ray, electrocardiogram, echocardiography, and chest computed tomography (CT) scan with image reconstruction to evaluate the cardiopulmonary function, condition of thoracic deformity, and presence of comorbidities.

### 2.1. Minimally Invasive Sternal Depression Procedure

**Figure 3:**
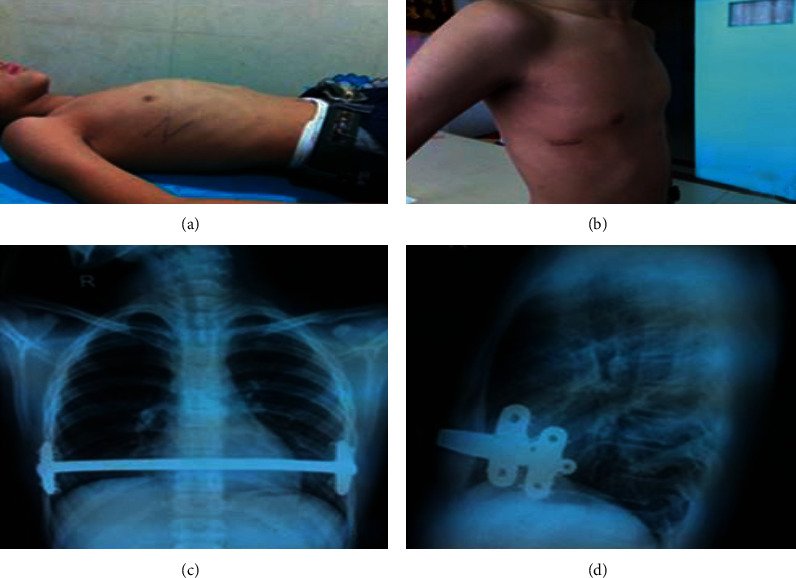
Minimally invasive sternal depression procedure for pectus carinatum: (a) preoperative appearance, (b) postoperative appearance and the incision locations, (c) postoperative frontal chest X-ray film, and (d) postoperative lateral chest X-ray film.

The patients laid in the supine position with both upper limbs abducted. A suitable type of steel bar was selected. The assistant pressed the protruding sternum until the desired height of the flat thorax was reached. Based on the shape of the chest at the time, the orthotic steel bar was bent into a bow shape. A 2.0 cm transverse incision was made at the midaxillary line on either side of the chest. The muscularis was dissociated to expose the ribs and intercostal muscles. The rib periosteum was dissociated. A steel wire was used to mount both ends of the Nuss bar stabilizer on the corresponding hard ribs and ensure that the stabilizers were parallel to the midaxillary lines. A tunnel was made at the subcutaneous tissue along the ribs and anterior to the sternum using a pair of long-curved tissue scissors. Damage to the pleura and stray into the thoracic cavity were avoided as far as possible. After the perforation of the tunnel anterior to the sternum, a chest tunneling tube with a pinhole was passed through the tunnel anterior to the sternum to the opposite side. The pinhole was retracted. The bent orthotic bar was inserted into the chest tube from one end and guided across the tunnel. The bar was then flipped over so that its back arch faced anteriorly. The sternum was pressed to a desired height, and a steel wire was used to fix both ends of the bar firmly with the stabilizers. The orthotic correction of the chest wall was completed (Figure 3).

### 2.2. Minimally Invasive Sternal Depression Procedure+Nuss Procedure

**Figure 4:**
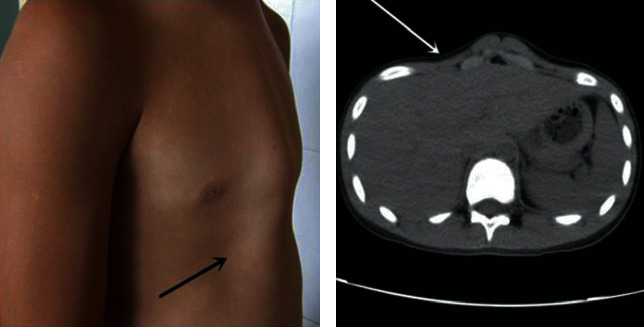
Pectus carinatum comorbid with depressed Harrison groove deformity (arrow indicates the Harrison groove depression site).

**Figure 5:**
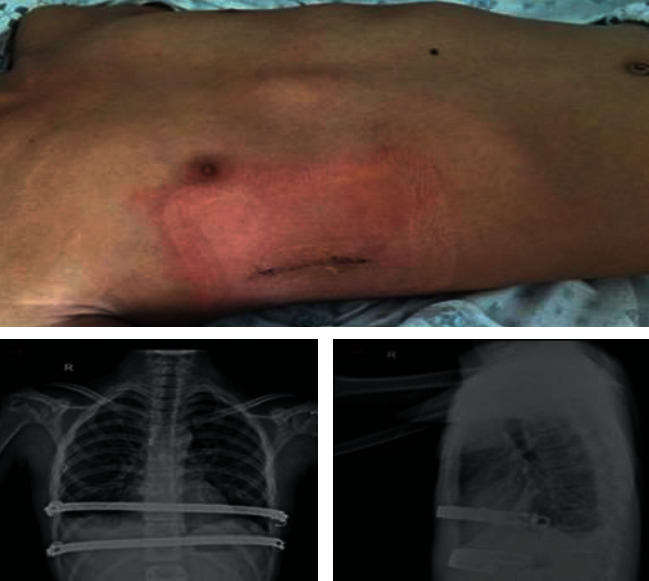
Pectus carinatum comorbid with depressed Harrison groove deformity after surgery (appearance of the chest and anterior and lateral chest X-ray films).

The surgical procedure was implemented to treat pectus carinatum comorbid with obvious Harrison groove depression. Upon completion of the routine minimally invasive sternal depression procedure, the sternum was depressed to a normal position. However, the depression of the Harrison groove was severe on either side of the chest. The minimally invasive Nuss procedure was performed at the lowest point of depression until a satisfactory orthotic effect was reached (Figures 4 and 5).

### 2.3. Transverse Sternal Osteotomy+Minimally Invasive Nuss Procedure

**Figure 6:**
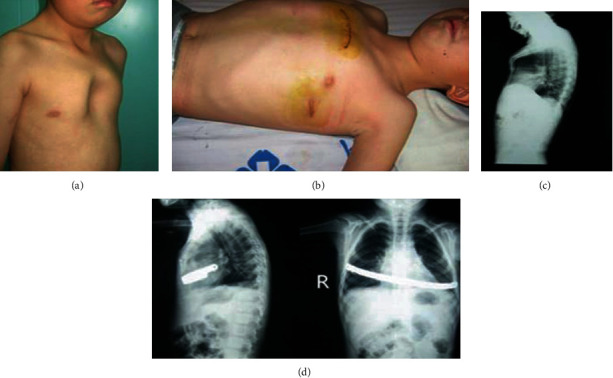
Pectus carinatum (pouter pigeon breast) comorbid with pectus excavatum: (a) preoperative appearance, (b) postoperative appearance and surgical incisions, (c) preoperative lateral chest X-ray film, and (d) postoperative anterior and lateral chest X-ray films.

The surgical procedure was implemented to treat a pouter pigeon breast comorbid with pectus excavatum. A transverse incision was made at the sternal protruding site, and the protruding sternum was wedge resected. The broken end of the sternum was sutured by a steel wire. The protruding costal cartilages were resected from the periosteum on both sides of the sternal protruding site. A polyester suture was used to reapproximate the broken ends of the costal cartilages. The Nuss procedure was implemented at the lowest level of the sternal depression site. The steel bar was inserted and flipped over to flatten the depressed chest wall (Figure 6).

### 2.4. Statistical Analysis

Excel 2010 was used to build the database. The IBM SPSS Statistics 23.0 software was used for statistical analysis. Quantitative data are described as the mean + standard deviation, and an intergroup comparison was performed using a one-way analysis of variance. Qualitative data are presented using the frequency, and an intergroup comparison was performed using a *χ*^2^ test, in which a difference of *P* < 0.05 was considered statistically significant.

## 3. Results and Discussion

**Figure 7:**
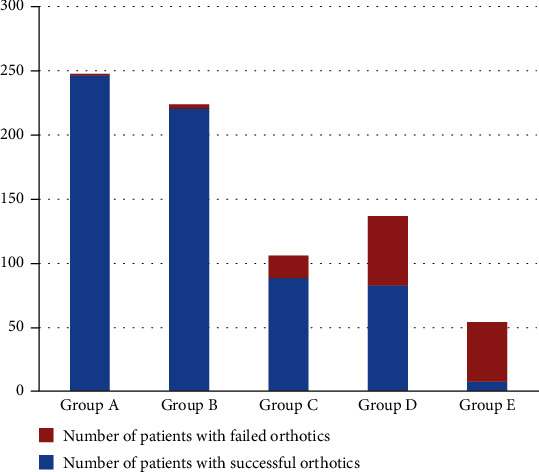


All 767 pediatric patients received orthotic bracing at first. Patients were divided into five groups according to their age: A (2.16–6 years), B (6–9 years), C (9–12 years), D (12–15 years), and E (15–18 years). Most pediatric patients (*n* = 644) obtained satisfactory chest appearance, and the success rate was 84.0%. There were 97 pediatric patients with an unsatisfactory orthotic effect and 26 who experienced a relapse of pectus carinatum shortly after correction. These two categories were considered orthotic failure. Among the 123 failure cases, 108 pediatric patients underwent minimally invasive surgery. There was a significant difference in the bracing failure rates among the five groups (*P* < 0.001; Figure 7). The orthotic effect of bracing was better among younger pediatric patients. The rate of orthotic bracing failure was correlated with age among pediatric patients (*P* < 0.001).

Among the 123 pediatric patients in whom orthotic bracing failed, 108 underwent minimally invasive sternal depression. For the 15 pediatric patients who did not receive the surgery, their chest appearance was continuously observed. All 108 pediatric patients successfully completed the surgery. A minimally invasive sternal depression procedure was used to treat 76 cases of pediatric patients with chondrogladiolar prominence. For the 29 comorbid cases of obvious Harrison groove depression, the minimally invasive sternal depression procedure+Nuss procedure was carried out. Transverse sternal osteotomy+minimally invasive Nuss procedure was used to treat two cases of pouter pigeon breast with pectus excavatum. For one case of neurofibromatosis, the minimally invasive sternal depression procedure+neurofibromatosis excision surgery was carried out. The average operation time was 112.8 ± 23.5 min. After the surgery, four cases presented with subcutaneous emphysema, three cases presented with incision wound infection, and one case presented with steel bar rejection responses. All pediatric patients were given an analgesic pump to relieve pain after surgery. Two days after surgery, the patients started to get out of bed, walk, and do some activities. The average length of hospitalization after the surgery was 7 days. The time for steel bar removal surgery was decided based on the development of the chest and changes in the position of the steel bar. Normally, after a period of approximately 2 years, the steel bar is removed. Currently, there have been 77 pediatric patients who have had their steel bars removed. A postoperative follow-up was performed at 1 month, 6 months, 1 year, and 2 years after the steel bar placement surgery. After the steel bar was removed, a follow-up was performed yearly for 3 years.

The treatment methods for pectus carinatum are divided into traditional surgery (Ravitch operation), minimally invasive sternal procedure, and orthotic bracing. The Ravitch sternal depression procedure and its modifications for pectus carinatum are implemented by an incision at the center of the chest to dissociate the pectoralis major on both sides and the removal of the deformed costal cartilage to achieve sternal depression [1]. The surgical incision is long, and resection of the costal cartilage is needed. It is highly invasive with slow recovery and can cause shrinkage of the pleural volume, thus affecting the cardiopulmonary function. This surgical method has been gradually replaced by other treatment methods. Inspired by the theory of the Nuss procedure for pectus excavatum, Abramson reported pectus carinatum treated by minimally invasive surgery in 2005 [7], and this method was rapidly promoted worldwide soon after. Compared with the traditional Ravitch sternal depression procedure and its modifications, the minimally invasive sternal depression procedure has the following advantages. The orthotic steel bar is placed into the tunnel formed at the subcutaneous tissue anterior to the sternum but not into the thoracic cavity. It avoids damage to the important organs in the thoracic cavity and does not affect the cardiopulmonary function. It is not restricted by pleural adhesion and reduces surgical risk. After the sternal depression procedure, the volume of the thoracic cavity is not reduced, benefiting the growth and development of pediatric patients. The operation time is short, and the wound is small. The incision is made at the lateral chest wall without incision at the center of the chest, providing good aesthetic effect. The recovery period is fast, resulting in short hospital stays. Thus, it can be easily accepted by pediatric patients and parents [8, 9]. In 1979, Haje and Raymundo reported their preliminary experience with dynamic compression bracing for chest orthotic treatment [10]. Thereafter, Dr. Martinez-Ferro and his team from Argentina developed the dynamic compression system [11], which is equipped with a pressure control detection system. By compressing toward the protruding sternum through long-term wearing of custom-made orthotic braces, it achieves the purpose of sternal remodeling. Since orthotic bracing treatment possesses advantages such as no requirement for surgery or hospitalization, risk associated with anesthesia, surgical complications, or surgical scar and a reduced cost of treatment, it is currently the first-line treatment method for pectus carinatum and has been widely promoted worldwide [2, 12, 13].

The principle of both orthotic bracing and the minimally invasive sternal depression procedure is the continuous exertion of force toward the sternum so that the protruding chest contracts inward to achieve the orthotic treatment effect. Since orthotic bracing does not cause wounding or require anesthesia or hospitalization, it should be the first-line treatment method of choice for pectus carinatum. However, it has been reported that the failure rate of orthotic bracing increases with age. This may be due to the sternum and ribs of older pediatric patients having a greater hardness and lower chest compliance, so the compressive force of the brace is insufficient to correct the deformity of the protruding sternum. Additionally, the compliance of pediatric patients to medical orders is low and can result in an insufficient duration of bracing, improper bracing, and dislocation of the compressor, which are the main factors contributing to the failure of orthotic correction. We requested an orthotic brace wearing time of more than 12 h daily, with longer brace wearing times with age. The treatment period was half a year. For older pediatric patients, the treatment period can be prolonged for a year or longer. To prevent a relapse, pediatric patients are usually requested to keep wearing the braces for 2–3 months after the flattening of the chest.

The surgical indications for minimally invasive sternal depression are summarized below [4], and it should include two or more of the following criteria: (1) a CT Haller index of <2.30; (2) pulmonary function test, electropherogram, and echocardiography tests demonstrating abnormal pathological changes such as abnormal cardiac function and restrictive or obstructive airway; (3) progressive deformity or comorbidity with obvious symptoms; and (4) the patient not accepting the appearance of the deformed chest. The key to a successful surgery is the measuring, shaping, placement location, and reliable fixation of a steel bar; otherwise, prolapse and displacement of the orthotic steel bar may occur as a severe postoperative complication. The orthotic steel bar and stabilizers, as well as the stabilizer and ribs, are strapped and stabilized by a steel wire. They bear a great force in compressing the sternum. No case presented with severe complications in this group, and all cases demonstrated satisfactory near- and long-term effects after surgery. Our experience and learning mainly included the following: (1) after dissociating the periosteum of ribs, a steel wire was inserted, and each hole of the stabilizer was mounted by two steel wires to prevent sliding along with the ribs; (2) the stabilizers were strictly ensured to be parallel to the midaxillary line for a uniform compressive force; (3) a thicker steel wire was selected to wind the distant ends of the orthotic steel bar a few times to avoid slipping; and (4) bending over, chest expansion, rolling, and strenuous activities of the upper limbs must be avoided after surgery. Other possible complications also included pneumothorax, hemothorax, and wound infection. Pneumothorax and hemothorax were mainly related to the damage of the pleura. While dissociating the subcutaneous tissue to create a tunnel, it should be manipulated anteriorly to the pectoralis major, as well as in the subcutaneous fat along the sternum and costal cartilage to prevent stray into the thoracic cavity. (5) When threading the steel wire, the periosteum of the ribs was dissociated and pushed aside first to avoid damage to the pleura. This also prevented damage to the intercostal blood vessels and intercostal nerves, reducing pain after surgery. (6) The development of the chest muscularis of pediatric patients was incomplete, and the chest wall was thinner. Once the wound was infected, the orthotic steel bar and stabilizers were removed and, in turn, caused surgery failure. The implanted steel bar, stabilizers, and steel wire should be covered and wrapped by the muscularis. Bleeding must be stopped completely before suturing to prevent the accumulation of blood and effusion and reduce the opportunity of wound infection.

There are also certain limitations of the minimally invasive sternal depression procedure. Since it can only compress the sternum, it cannot correct costal cartilage deformities. Hence, it is only suitable for treating chondrogladiolar prominence. For comorbidity and chondromanubrial prominence cases, a flexible individualized surgical method should be implemented. In clinical practice, there were some pediatric patients with a wide scope of pectus carinatum, for whom a steel bar could hardly achieve satisfactory orthotic effects, and two to three steel bars needed to be inserted. Some pediatric patients had pectus carinatum comorbid with severe Harrison groove depression. During the correction of the protruding sternum by orthotic bracing or minimally invasive sternal depression, a more severe depression of the Harrison groove often occurred, which affected the appearance after correction. For these patients, the correction method of the minimally invasive Nuss procedure for pectus excavatum should be carried out at the lowest point of depression. A steel bar should be inserted inferior to the depression of the sternum to raise the depressed Harrison groove to achieve satisfactory orthotic effects. Some other cases of pouter pigeon breast comorbid with pectus excavatum were often treated by the minimally invasive Nuss procedure combined with osteotomy to achieve a satisfactory orthotic effect.

## 4. Conclusions

In summary, although some pediatric patients do not have significant symptoms, pectus carinatum will affect their psychological development, and the deformity is likely to present with progressive aggravation along with age. Therefore, after the diagnosis of pectus carinatum is confirmed, patients should be actively treated, and early treatment has a better orthotic effect. Noninvasive orthotic bracing is the treatment of choice, and for pediatric patients who fail orthotic bracing, a minimally invasive sternal depression procedure can be selected as the treatment. The study confirmed that the older the patients, the higher the failure rate of orthotic effect. Minimally invasive surgery should be considered the preferred treatment for pectus carinatum over 15 years old. For pediatric patients with chondromanubrial prominence, minimally invasive sternal depression often cannot achieve satisfactory surgical outcomes. An individualized surgery plan should be carried out. Problems such as selecting the length of the steel bar, insertion location, number of steel bars, and whether to combine with a transverse osteotomy or the minimally invasive Nuss procedure should be planned in detail before surgery to achieve satisfactory orthotic effects.

## Data Availability

This manuscript has not been published or presented elsewhere in part or in entirety and is not under consideration by another journal; all basic data is available. The need for informed consent was waived, and the study design was approved by the appropriate ethics review board. We have read and understood your journal's policies, and we believe that neither the manuscript nor the study violates any of these. There are no conflicts of interest to declare.
